# Innate immune response after adenoviral gene delivery into skin is mediated by AIM2, NALP3, DAI and mda5

**DOI:** 10.1186/2193-1801-2-234

**Published:** 2013-05-24

**Authors:** Matthias Schulte, Michael Sorkin, Sammy Al-Benna, Jadwiga Stupka, Tobias Hirsch, Adrien Daigeler, Marco Rainer Kesting, Hans-Ulrich Steinau, Frank Jacobsen, Lars Steinstraesser

**Affiliations:** Laboratory for Molecular Oncology and Wound Healing, Department of Plastic Surgery, BG University Hospital Bergmannsheil, Ruhr University Bochum, Bochum, Germany; Department of Oral and Maxillofacial Surgery, Klinikum Rechts der Isar, Technical University Munich, Munich, Germany

**Keywords:** Adenovirus, Keratinocytes, Skin, Gene therapy, Innate immunity, Signal transduction

## Abstract

Methods for human skin gene therapy requires efficient and stable introduction of genes into skin cells. Transient cutaneous gene therapy is an attractive approach in the treatment of skin diseases. The ‘Achilles heel’ of adenoviral gene therapy is its immunogenicity and many aspects of adenovirus induced cutaneous immune reaction still remain unanswered, particularly the role of keratinocytes. Therefore, human keratinocytes were transfected with adenoviral DNA and cytokine expression was analyzed. Moreover, adenoviral transduction of full-skin was performed *ex vivo* and *in vivo*. We observed cytokine induction after cytoplasmatic internalization of adenoviral DNA into epidermal cells. Inhibition of AIM2, NALP3, DAI or mda5 downregulated the cytokine response. Transduction of immunocompetent mice led to a detectable transgene expression for 12 days. Re-application of the vector led to a decrease in intensity and duration of transgene expression limited to 4 days and an increased cytokine expression. In contrast, immunodeficient mice showed a reduced expression of cytokines after DNA internalization. AIM2, NALP3, DAI and mda5 are essential in the induction of an innate immune response towards adenoviral DNA. This immune reaction leads to a decrease in transduction efficiency of the vector after re-application and modulation of these receptor systems stabilizes transgene expression.

## Introduction

The skin is the biggest and most important organ in protecting the body from a hostile environment. The epidermis, its outside layer, is mainly composed of keratinocytes, which guard the body against physical, chemical, or biological damage by establishing a protective layer ( Bouwstra et al. 2003 ). Its accessibility makes the skin an easily approachable target for the treatment of both local and systemic diseases via gene therapy ( Kim et al. 2000 ). Gene therapy is a promising tool for the treatment of a wide variety of inherited as well as acquired disease including genetically inherited skin disorders, tumors, metabolic disorders and infectious diseases ( Mulligan 1993 ). Specific anatomical and biological properties make the skin a very interesting organ for *in vivo* and *ex vivo* gene therapy approaches. Gene delivery can be easily controlled and the skin surgically excised if any side effects occur ( Christensen et al. 2001 ). Keratinocytes are responsible for establishing a physical barrier and guaranteeing the structural integrity of the epidermis ( Bouwstra et al. 2003 ). As the epidermis is known to produce a variety of cytokines and growth factors, keratinocytes may also be engineered as bioreactors to secrete gene products, which have local or systemic effects ( Tomic-Canic et al. 1998 ). Its accessibility suggests that different methods for gene delivery can be pursued, depending on the desired application. The approach used to deliver DNA into the skin will have an influence not only on the efficiency of DNA delivery, but also on the level and duration of transgene expression ( Worgall et al. 1997 ). For transient transduction of target cells, adenoviral vector systems possess the highest efficacy and have been used in 23.3% of the registered clinical trials worldwide in the last two decades ( JGT 2012 ).

*Adenoviridae* are non-enveloped, double stranded (ds), linear desoxyribonucleic acid (DNA) viruses with a genome of 35–40 kb and a particle size of 70–100 nm ( Rux & Burnett 2004 ). The adenoviral genome is well characterized and comparatively easy to manipulate. Most adenoviruses cause mild diseases in immunocompetent human adults and by deletion of crucial regions of the viral genome the vectors can be rendered replication defective, which increases their predictability and reduces unwanted side effects. Moreover, deleted regions of the viral genome can easily be replaced by foreign genomic material encoding the therapeutically active metabolite ( Tatsis & Ertl 2004 ). The process of adenoviral entry into the host is extremely efficient and has been intensively studied ( Douglas 2007 ). Adenoviruses exhibit a wide host range *in vitro* and *in vivo*; this range was also seen in nondividing cells ( Zhang 1999 ). In addition, the well-defined and easily manipulated viral genome favors the development of adenoviral vectors for gene therapy applications. A major disadvantage of adenoviral vectors is that viral DNA can effectively elicit the innate and adaptive immune response immediately after infection, leading to the secretion of pro-inflammatory cytokines in mice, primates and humans ( Raper et al. 2003 ; Schnell et al. 2001 ; Zhang et al. 2001 ). Activation of innate immunity is associated with a reduction in efficacy of gene transfer ( Worgall et al. 1997 ) but also in profound damage to healthy tissue and significant morbidity in transduced hosts ( Raper et al. 2003 ; Schnell et al. 2001 ). Several studies focused on the immune reaction elicited through cytoplasmatic localized adenoviral DNA. This led to the development of newer generations of adenoviral vector systems that were depleted of a number of viral genes in order to reduce the immune reaction. Helper-dependent adenoviral vectors lack almost all viral coding sequences and lead to diminished adaptive immune responses and improved duration of gene transfer ( Muruve 2004 ). However, acute toxicity and reduced vector persistence provoked by the innate immune response remain the most significant barriers to an effective clinical application of this promising technology ( Brunetti-Pierri et al. 2004 ).

Several studies on adenoviral DNA induced innate immune reaction have focused on antigen presenting cells (APCs) such as dendritic cells (DCs) or macrophages (MΦ) ( Nociari et al. 2007 ; Zhu et al. 2007 ) and RNA virus-induced immune reactions of APC ( Lopez et al. 2006 ). In addition to adenoviral DNA, activation of innate immunity has also been described for vertebrate and mammalian DNA, and synthetic oligonucleotides ( Nociari et al. 2007 ; Zhu et al. 2007 ). Moreover, a sequence independent mechanism for cytoplasmatic DNA recognition and immune activation has been specified ( Suzuki et al. 1999 ). The detection of microbial components by pattern recognition receptors (PRRs) is one of the earliest defense mechanisms to trigger innate immune responses against infections ( Janeway & Medzhitov 2002 ). Of the many classes of molecules detected by cells as pathogen associated molecular patterns (PAMPs), nucleic acids are potent and broadly recognized ( Isaacs et al. 1963 ). In order to sense nucleic acids the immune system employs several classes of receptors. The family of Toll-like receptors (TLRs) in this context is the best described group of PRRs. TLRs can recognize endosomal doublestranded (ds)RNA (TLR-3) ( Alexopoulou et al. 2001 ), singlestranded (ss)RNA (TLR-7/-8) ( Diebold et al. 2004 ), or hypomethylated DNA (TLR-9) ( Hemmi et al. 2000 ). Activation of nucleic acid sensing TLRs occurs within endosomal compartments (TLR-3, -7, -8 and −9) and requires either a myeloid differentiation primary response gene 88 (MyD88) or TIR-domain-containing adapter-inducing interferon-β (TRIF) adapter molecules. These proteins facilitate activation of downstream signaling cascades, which lead to the activation of inflammatory transcription factors, including nuclear factor-kappa B (NFκB), activator protein 1 (AP-1) and interferon regulatory factors (IRF) 3 and 7. An activation of downstream cascades leads to a release of inflammatory cytokines which play an inportant role in direct or indirect viral clearing mechanisms. A direct mechanism is associated with a recruitment of inflammatory cells, activation of adaptive immune system and complement system whereas an indirect mechanism is represented by a further induction of inflammatory signalling, for example an activation of JAK/STAT-signalling. The JAK/STAT-signal cascade is activated mainly through cytokine receptors on the cell surface and thereby plays during an infection in the communication between cells play a central role. An activation of this signaling cascade leads to further induction of cytokine synthesis in the cell ( Kanehisa 2012 ).

Since the discovery of TLR-9, there has been a growing body of evidence that DNA derived from microbial and host cells can be recognized via a TLR-9-independent mechanism. DNA recognition in these pathways is sequence independent and occurs in the cytoplasm of the cells ( Suzuki et al. 1999 ). Different ligands, such as adenoviral, mammalian and vertebrate DNA as well as dsDNA have been characterized for TLR-independent recognition in APCs ( Martin & Elkon 2006 ; Nociari et al. 2007 ; Zhu et al. 2007 ). In addition to TLRs, the RIG-like receptor (RLR) mediated signal transduction has also been discussed for DNA-recognition wherein a corresponding DNA-sensing receptor in this signaling cascade has not been identified yet ( Nociari et al. 2007 ). RLR-dependent signaling is induced by recognition of cytosolic ribonucleic acid (RNA) via retinoic acid-inducible gene I (RIG-I) and melanoma differentiation associated gene 5 (mda5)) and requires an adapter molecule IPS-1 (mitochondrial antiviral signaling protein 1). Downstream signaling results in an NFκB-, AP-1- and IRF-3/7-dependent induction of cytokine expression ( Yoneyama & Fujita 2007 ). In 2009, a DNA sensor and activator of innate immune responses has been identified and termed DNA-dependent activator of IFN-regulatory factors (DAI and also known as DLM-1 and ZBP1) ( Takaoka et al. 2007 ). The activation of DAI leads to a RIP1/3-dependent activation of NFκB. Hence, an activation of IRF-3 and IRF-7 has also been reported ( Kanehisa 2012 ). Subsequent studies have shown the presence of an additional mechanism(s) for DNA-sensing and activation of the innate immune system. NACHT-leucine-rich repeat-PYD containing protein 3 (NALP3) also known as cryopyrin, and its adaptor protein apoptosis-associated speck-like protein containing a CARD (ASC), regulate secretion of interleukin (IL)-1β in response to an adenovirus infection. Inflammasome activation also occurs upon cytosolic exposure of DNA, though in different other reports DNA-sensing was shown to be dependent on ASC and not NALP3 ( Muruve et al. 2008 ). In particular a group of proteins of the HIN-200 (hematopoietic interferon-inducible nuclear proteins with a 200-amino-acid repeat) protein family, which exhibit a DNA-binding domain along with a CARD-domain, became more and more important in the last years. For this protein family, an induction of innate immunity dependent of AIM2 (absent in melanoma 2) and IFI16 (gamma-interferon inducible protein 16) via ASC has been described ( Roberts et al. 2009 ; Unterholzner et al. 2010 ).

However, data on the role of epithelial cells in innate immunity, particularly in response to DNA internalization and DNA virus infection is limited. Also, there is a lack of information about constitutive expression of inflammatory factors in keratinocytes, as well as data about the induction of inflammatory factors after adenoviral DNA internalization still are missing. On the way to improve safety, efficacy and duration of cutaneous adenoviral gene therapy it is necessary to get a basic knowledge on the reaction of epidermal cells in response to adenoviral gene delivery. This study was performed in order to depict the important role of human epidermal cells in innate immunity towards adenoviral vector systems. This will help to fine tune various therapeutic intervention strategies. Therefore, this study observed the mechanisms of innate immune reaction of cultured keratinocytes *in vitro*, the immune response to adenoviral gene delivery into human skin samples *ex vivo* by using a human full skin culture system ( Steinstraesser et al. 2009 ) and *in vivo*, using a murine transduction model.

## Materials and methods

### Keratinocyte cell culture

Fresh human skin was obtained after abdominoplasty surgery (informed consent was given by the patient) and washed in PBS (PAA Laboratories, Coelbe, Germany). The skin was placed in a sterile petri dish and the hypodermis was excised. The skin was disinfected with Lavasept (Braun AG, Melsungen, Germany) for 5 min and washed with PBS, the tissue was sliced into pieces of 1 cm^2^. Skin pieces were transferred into a new petri dish with the epidermal side up and the skin was completely immersed with freshly prepared 0.2% dispase-solution (4.7 U/ml, Gibco, Paisley, United Kingdom [UK]) and incubated overnight at 4°C. The epidermis was peeled off and placed in Trypsin/EDTA-solution (0.05%/0.02%, Gibco, Paisley, UK) and reduced to pieces as small as possible. The pieces were incubated at 37°C for 20 min in a gently shaking (180 rpm) waterbath (GFL Burgwedel, Germany). The cell suspension was vortexed and the trypsin digestion was stopped by adding fetal bovine serum (FBS, HyClone, Logan, USA). The suspension was filtered through a 100 μm cell strainer (Becton Dickinson Heidelberg, Germany) and centrifuged at 400 × g, 20°C for 5 min. The cells were resuspended in a 5 ml keratinocyte medium (containing 3:1 Dulbecco’s Modified Eagle Medium (DMEM, Gibco, Paisley, UK), Ham’s F12 (Gibco, Paisley, UK), 10% FBS (Hyclone, Logan, USA), 1% Penicillin/Streptomycin (ICN, Aurora, USA), 4 mM L-Glutamin (ICN, Aurora, USA), 24.3 μg/ml Adenine (Calbiochem, Darmstadt, Germany),5 μg/ml Insulin (Sigma, St. Louis, USA), 0.8 μg/ml Hydrocortisone (Calbiochem, Darmstadt, Germany), 1.346 ng/ml Triiodothyronine (Sigma, St. Louis, USA), 1 μM Isoproterenol (Sigma, St. Louis, USA), 20 ng/ml hEGF (Sigma, St. Louis, USA) and counted by CASY-1 (Schärfe-System, Reutlingen, Germany). Cells were seeded at a density of 75,000 cells/cm^2^ into collagen type I (Becton Dickinson Falcon, Heidelberg, Germany) precoated culture flasks. All different cell types including HaCaT (kindly provided by Prof. Fusenig, University of Heidelberg) cell lines were cultured at 37°C in humidified atmosphere of 5% CO_2_. HaCaT cells were cultured in DMEM containing 10% FBS (Hyclone, Logan, USA) and 1% Penicillin/Streptomycin. Medium was changed every second day.

### Human full skin culture

Fresh, sterile human skin explants were obtained in the operating room of the University Hospital Bergmannsheil, Bochum, Germany from six adult healthy patients (six different donors; age range: 19–43 years) undergoing abdominoplasty surgery. The study was approved by the local ethics committee (registration number: 2501; institutional review board of the Faculty of Medicine, Ruhr-University Bochum), and all of the patients gave written informed consent.

Skin explants were cultured as described by Steinstraesser et al. ( Steinstraesser et al. 2010 ). For transduction, 10^10^ infectious units (IU) Ad-GFP (green fluorescent protein) in 50 μl PBS were intradermally injected (n = 6 samples from two different donors per group). 3 to 96 h post transduction, tissue biopsy specimens were harvested for total RNA isolation. Transgene expression was localized via Kodak Imaging Station 4000MM and Kodak MI software.

### Production and purification of recombinant adenovirus

A replication-deficient human *Δ*E1 adenovirus type 5 (Ad5) with inserted cytomegalovirus (CMV)-promoter driven green fluorescent protein (GFP), *Δ*E1-Ad5-CMV-GFP, was used as described by Steinstraesser et al. ( Steinstraesser et al. 2011 ).

**DNA purification, RNA isolation and Reverse Transcription** were performed as described by Steinstraesser et al. ( Steinstraesser et al. 2011 ).

### Transfection

Cells were grown in 6-well plates until 90-100% confluency. DNA transfection complexes were prepared according to the manufacturer’s instructions (Roche Molecular Biochemicals, Mannheim, Germany). Briefly, *Δ*E1-Ad5-CMV-GFP DNA was mixed in ratio of 2:5 with the Fugene® HD transfection reagent (Roche Molecular Biochemicals, Mannheim, Germany) in PCR-grade water (Roche Molecular Biochemicals, Mannheim, Germany) for 15 min at room temperature and then added to cells. If not mentioned otherwise, all transfection experiments were performed in triplicate for each group.

For siRNA transfection, keratinocytes were sown in a density of 15.000 cells/cm^2^ (HaCaT) or 35.000 cells/cm^2^ (HKC) in 12-well cell culture plates. After 24 hours the medium was changed and 400 μl serum-reduced medium. The siRNA transfection complex was generated by combining siRNA (Eurofins MWG Operon, Ebersberg, Germany) and X-tremeGENE® siRNA Transfection Reagent (Roche, Mannheim, Germany) in Opti-MEM (PAA Laboratories, Coelbe, Germany) according to manufacturer’s instructions (ratio of gene X-tremeGENE®: siRNA = 1: 0.2). Before DNA transfection, the cells were cultivated for another 48 hours with siRNA-transfection complexes. Specific siRNA sequences are listed in Table 1.

**Table 1:** **List of oligonucleotides used for siRNA mediated gene silencing**

Gene	Sequence
AIM2-NM_004833.1	5′-GCACCAUAAAGGUUAUUAA-3′
NALP3-NM_004895.4	5′-GCUUUGUCCUCGGUACUCA-3′
mda5-NM_020746.4	5′-GGAAUAAUCUUUACAAAAA-3′
DAI-NM_030776.2	5′-CAAAAGAUGUGAACCGAGA-3′
Control	5′-AGGUAGUGUAAUCGCCUUG-3′

### Protein inhibition

Prior to transfection, cells were pretreated with specific inhibitors (10 μM each) for NFκB (IκB–α inhibitor BAY117082 in DMSO), ERK1/2 (PD98059 in DMSO), p38 MAPK (SB203580 in DMSO), JNK (JNK II–inhibitor SP600125 in DMSO) and JAK-STAT (AG490 in EtOH) according to manufacturers instructions (Sigma, Steinheim, Germany).

### Real-time PCR

Relative Quantification of mRNA was performed in a two-step real-time RT-PCR procedure using the fluorescent dye SYBR Green I (Light Cycler FastStart DNA Master SYBR Green I, Roche, Mannheim, Germany) and a Light Cycler 480 (Roche, Mannheim, Germany). The first step consisted of an RT reaction as described above, the second step of PCR amplification with specific primers listed in Table 2. These primer pairs were validated to generate a single PCR-product. The PCR reactions were performed with 2 μl of cDNA, 0.5 μM of sense and antisense primers, 3 mM MgCl_2_ and 2 μl of FastStart SYBR Green reaction mix in a total volume of 20 μl. The cycling conditions were as follows: 95°C for 10 min at a ramp speed of 20°C/sec, 40 cycles (if not differently described) consisting of 94°C for 15 sec at a ramp speed of 20°C/sec, A primer specific annealing temperature (Table 1) for 10 sec at a ramp speed of 20°C/sec, 72°C for 10 sec at a ramp speed of 20°C/sec, followed by a melting point analysis: 95°C for 0 sec at a ramp speed of 20°C/sec, 65°C for 15 sec at a ramp speed of 20°C/sec, 95°C for 0 sec at a ramp speed of 0.1°C/sec, and finally a cooling phase: 40°C for 30 sec at a ramp speed of 20°C/sec. mRNA concentrations were corrected to 18S rRNA in each sample and were normalized to an untreated control (x-fold expression).

**Table 2:** **List of oligonucleotides used for RT-PCR (h: human; m: murine; f: forward primer; r: reverse primer; TG: target gene; AT: annealing temperature)**

Target gene (TG)			Sequence [5′-3′]	AT[°C]
Interferon α (human) - NM_024013.2	hIFN-α1	f:	acccacagcct ggataacag	60
		r:	ctctcctcctgcatcacaca	
Interferon β (human) - NM_002176.2	hIFN-β	f:	actgcctcaaggacaggatg	60
		r:	agccaggaggttctcaacaa	
Interleukin 1α (human) - NM_000575.3	hIL-1α	f:	aatgacgccctcaatcaaag	60
		r:	tgggtatctcagqcatctcc	
Interleukin 6 (human) - NM_000600.3	hlL-6	f:	caatgaggagacttgcctgg	63
		r:	gcacagctctggcttgttcc	
Interleukin 8 (human) - NM_000584.3	hIL-8	f:	tctgcagctctgtgtgaagg	63
		r:	aatttctgtgttggcgcagt	
Tumor necrosis factor α (human) - NM_000594.3	hTNFα	f:	aacctcctctctgccatcaa	62
		r:	ggaagacccctcccagatag	
18S ribosomal RNA (human) - X03205.1	hl8S	f:	gaaaatgcgaatggctcattaaa	60
		r:	cacagttatccaagtaggagagg	
Toll-like receptor 3 (human) - NM_003265.2	hTLR-3	f:	agccttcaacgactgatgct	60
		r:	tttccagagccgtgcta.agt	
Toll-like receptor 7 (human) - NM_016562.3	hTLR-7	f:	ccacaaccaactqaccactg	60
		r:	ccacca.gacaaaccacacag	
Toll-like receptor 8 (human) - NM_138636.4	hTLR-8	f:	gtttcctcgtctcgagttgc	60
		r:	tcaaaggggtttccgtgtag	
Toll-like receptor 9 (human) - NM_017442.3	hTLR-9	f:	cctattcatggacggca.act	60
		r:	gagtgacaggtgggtgaggt	
Myeloid Differentiation Primary Response Gene 88 (human) - NM 001172567.1	hMyD88	f:	tgcagagcaaggaatgtgac	60
		r:	aggatgctggggaactcttt	
Interferon Regulatory Factor 3 (human) - NM 001571.5	hIRF-3	f:	qaggtgacagccttctaccg	60
		r:	tgcctcacgtagctcatcac	
Interferon Regulatory Factor 7 (human) - NM 001572.3	hIRF-7	f:	taccatctacctgggcttcg	60
		r:	gctccataaggaagcactcg	
Melanoma Differentiation-Associated Gene 5 (human) - NM_020746.4	hMda-5	f:	ggggcatggagaataactca	60
		r:	tgcccatgttgctgttatgt	
NACHT, LRR and PYD Domains-Containing Protein 3 (human) - NM 004895.4	hNALP3	f:	cttctctgatgaggcccaag	60
		r:	gcagcaaactggaaaggaag	
Retinoic Acid inducible Gene l(human) - NM 014314.3	hRIG-I	f:	gcaacagtgcagaggtgaaa	60
		r:	caaaagagcatccagcaaca	
Interferon α (murin) - NM_010502.2	mIFN-α	f:	tcaatgacctgcaagctgtc	62
		r:	agcaattggcagaggaagac	
Interferon β (murin) - NM_010510.1	mIFN-β	f:	ccctatggagatgacggaga	60
		r:	ctgtctgctggtggagttca	
Interleukin 1α (murin) - NM_010554.4	mIL-1α	f:	gcaacgggaagattctgaag	62
		r:	tgacaaacttctgcctgacg	
Interleukin 6 (murin) - NM_031168.1	mIL 6	f:	ccggagaggagacttcacag	60
		r:	tccacgatttcccagagaac	
Tumor necrosis factor α (murin) - NM_013693.2	mTNFα	f:	ccgatgggttgtaccttgtc	60
		r:	cggactccgcaaagtctaag	
18S ribosomal RNA (murin) - NR_003278.3	m18S	f:	cgcggttctattttgttggt	60
		r:	agtcggcatcgtttatggtc	
Green Fluorescent Protein - L29346.1	GFP	f:	acgtaaacggccacaagttc	60
		r:	aagtcgtgctgcttcatgtg	
DNA- Dependent Activator of Interferon- Regulatory Factors (human) - NM 030776.2	hDAI	f:	aaag catggacgattta ccg	60
		r:	atgatgttcccgtgtccaat	
Interferon, gamma- inducible Protein 16 (human) - NM 001206567.1	hIFI16	f:	gctgaccgaaacatggagat	60
		r:	cagatctcaactccccggta	
Interleukin 1β (human) - NM_000576.2	hIL-1β	f:	ttcgacacatgggataacga	60
		r:	tctttcaacacgcaggacag	
TIR- Domain-containing Adapter-inducing Interferon-β (human) - AB093555.1	hTRIF	f:	caggagcctgaggagatgag	60
		r:	ctgggtagttggtgctggtt	
Interferon-beta Promoter Stimulator 1 (human) - AB232371.1	hIPS-1	f:	ataagtccgagggcaccttt	60
		r:	gtgactaccagcacccctgt	
Abscent In Melanoma 2(human) - NM_004833.1	hAM2	f:	gctgcaccaaaagtctctcc	60
		r:	tcaaacgtgaagggcttctt	
Caspase-l (human) - NM_033292.3	hCASP-1	f:	gaaggcatttgtgggaagaa	60
		r:	ggtgtggaagagcagaaagc	

### Animal studies

The research protocol described below conformed to all regulations related to animal use and other German federal statues. It was performed in compliance with the ‘Guide for the Care and Use of Laboratory Animals’ associated with the German Animal Welfare Act. The animals were housed at an ambient temperature of 20 ± 2°C and on a 12: 12 h light/dark cycle. Both food and water were available *ad libitum*. Athymic mice (Foxn1^nu^) were obtained from Harlan Winkelmann (Borchen, Germany) and immunocompetent mice (SKH-1^h/r^) were obtained from Charles River (Wilmington, Sulzfeld, Germany). In the first experiment, immunocompetent mice were randomized into three groups with n = 3 mice. Each animal was intradermally transduced with 10^8^ - 10^10^ IU Ad-GFP in 50 μl PBS at two discrete areas on the back. Transgene expression was localized and quantified every second day via Kodak Imaging Station 4000 MM and Kodak MI software. 14 and 28 days after the first injection a second and third virus application was administered into the same areas and into a non-treated area.

In the second experiment, SKH-1^h/r^ and Foxn1^nu^ mice were each divided into six groups with n = 3 mice. Four distinct areas at the back of each mouse were marked. On day 0, 10^10^ IU Ad-GFP in 50 μl PBS or PBS alone were injected intradermally into two areas per mouse followed by a second injection of 10^10^ IU Ad-GFP into all four areas on day 14. 1, 6, 24, 48, 72 and 120 h after the second injection, one group was euthanized by intraperitoneal injection of 0.5 ml T61 (MSD Animal Health GmbH, Luzern, Switzerland), the transduced skin areas were excised and snap frozen in liquid nitrogen for RNA isolation.

### Statistical analysis

Differences were analyzed for statistical significance with the student’s *t*-test. Error bars represent standard errors of the mean (SEM). RT-PCR analysis was displayed as expression ratio of treated and untreated (vehicle control) cells (x-fold expression).

## Results

### Applicability of HaCaT cells and primary human keratinocytes (HKC)

Initially baseline expression of potential key molecules of the innate immune reaction after adenoviral transduction have been analyzed in primary human keratinocytes (HKC) and a keratinozyte cell line (HaCaT) via qRT-PCR (n = 6 samples).

**Figure 1:**
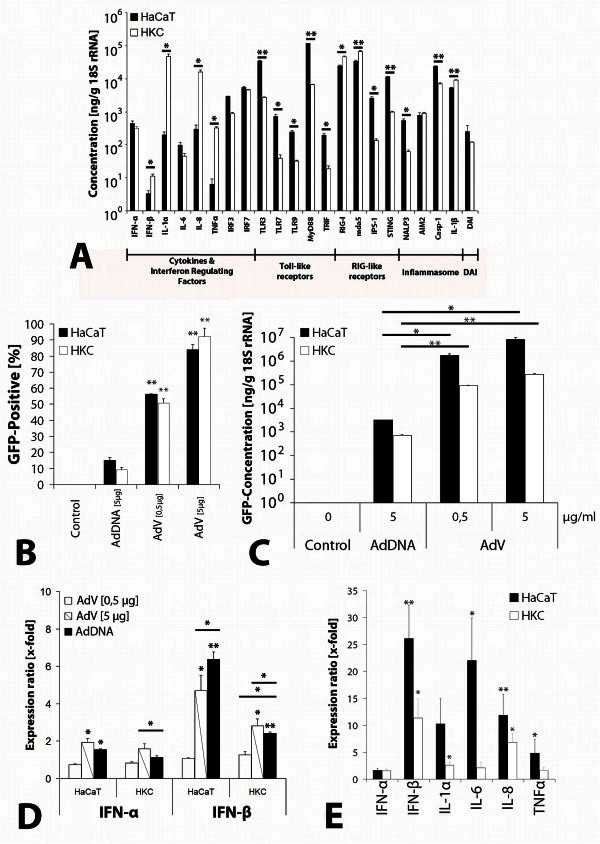
**Cutaneous adenoviral gene delivery.** (**A**) Baseline expression of cytokines and potentially in cytoplasmatic nucleic acid recognition involved receptors or adapter molecules in HaCaT cells and primary human keratinocytes (HKC). Data was generated via qRT-PCR and is displayed as mRNA concentration in ng/g 18S rRNA (* = p < 0,05; ** = p < 0,005). (**B**) Comparative efficiency of adenoviral vectors in HaCaT cells amd HKC. Quantitative analysis of GFP-positive cells 48 hours after transfection or transduction of HaCaT cells and HKC with 5 μg adenoviral DNA (AdDNA_[5μg]_) or 1.1 × 10^9^ IU (AdV_[0,5μg]_) and 1.1 × 10^10^ IU (AdV_[5μg]_) adenoviral vectors (* = p < 0,05; ** = p < 0,005). (**C**) Comparative GFP expression in HaCaT cells and HKC. The specific transcript was determined 48 h after transfection with 5 μg/ml medium of isolated adenoviral DNA (AdDNA_[5μg]_) and compared to transduction with 1.1 × 10^9^ IU (AdV_[0,5μg]_) and 1.1 × 10^10^ IU (AdV_[5μg]_) of a GFP-encoding adenoviral vector (* = p < 0,05; ** = p < 0,005). (**D**) Comparative type-I-interferon expression in HaCaT cells and HKC. The specific transcript was determined 6 h after transfection with 5 μg/ml medium of isolated adenoviral DNA (AdDNA_[5μg]_) and compared to transduction with 1.1 × 10^9^ IU (AdV_[0,5μg]_) and 1.1 × 10^10^ IU (AdV_[5μg]_) of a GFP-encoding adenoviral vector (* = p < 0,05; ** = p < 0,005). (**E**) Comparative cytokine expression after transfection of HaCaT cells and HKC with 5 μg/ml isolated adenoviral DNA for 15 h (HaCaT) or 6 h (HKC). These time points were determined (maximum expression) by a time course of cytokine expression in hacats in a previous study by Steinstraesser et al. ( Steinstraesser et al. 2011 ). Study groups included n = 18 (type-I-IFN) or n = 6 (cytokines) samples. Data was normalized to a vehicle control (* = p < 0,05; ** = p < 0,005).

The data analysis showed a clearly detectable expression of type-I-interferons (IFN-α and-β), interleukins (IL-1α,-1β and −6), chemokines (IL-8), tumor necrosis factor (TNFα) and interferon-regulatory factors (IRF-3 and −7) in HaCaT cells and HKC (Figure 1A). Even members of the Toll-like receptor family (TLR-3, -7, -9, MyD88 and TRIF), the RIG-like receptors (RIG-I, mda5, IPS-1 and STING) and NOD-like receptors (NALP3, AIM2 and caspase-1) and the DNA-binding protein DAI could be detected in both cell types. Based on these data, both cell types show an applicability for the following experiments. HaCaT cells, however, showed a significant higher mRNA expression in nine out of twenty-two genes measured. Apart from the basic applicability of both cell types, these differences in cytokine expression may influence the potential for a direct comparison of the data generated in the following experiments.

### Comparative analysis of transfection and transduction efficacy in keratinocytes

In order to determine the transduction efficacy of the used vector, keratinocytes were transfected/transduced with 5 μg of isolated DNA and an equivalent dose of adenoviral vectors (1.1 × 10^10^ infection units (IU)). A quantitative analysis of GFP-positive cells (Figure 1B) showed a transfection efficiency of 15% in HaCaT cells and 9% for HKC. Interestingly, transduction of keratinocytes with 1.1 × 10^9^ IU Ad-GFP (= 0.5 μg DNA) resulted in a significantly larger number of GFP-positive cells compared to transfection with 5 μg DNA (HaCaT: 56% (p = 0.0002); HKC: 51% (p = 0.0032)). Highest efficiency was obtained using 1.1 × 10^10^ IU Ad-GFP (HaCaT: 84% (p = 0.0138); HKC: 92% (p = 0.0055)), suggesting a concentration dependence in the transduction efficiency.

In terms of GFP mRNA expression, a concentration of 3.2 μg/g 18S rRNA (HaCaT, p = 0.0009) and 0.7 μg/g 18S rRNA (HKC, p = 0.01) were detected in cells treated with adenoviral DNA (Figure 1C). In contrast, keratinocytes that were transduced with 1.1 × 10^9^ IU of adenoviral vectors, showed a 550-fold (HaCaT, p = 0.0295) or 131-fold (HKC, p = 0.0070) higher levels of GFP mRNA. A transduction of cells with 1.1 × 10^10^ IU Ad-GFP led to a 2580-fold (HaCaT, p = 0.0402) or 398-fold (HKC, p = 0.0018) higher mRNA concentration in comparison to transfected cells. In addition, a significantly higher concentration of GFP mRNA was measured in cells treated with 1.1 × 10^10^ IU Ad-GFP in comparison to keratinocytes treated with 1.1 × 10^9^ IU (HaCaT: p = 0.0296; HKC: p = 0.0071).

### Comparative analysis of type-I-interferon expression

Type-I-interferons play a key role in the induction of immune responses to viral infection und are induced immediately after detection of viral components in the host cell. In addition to its direct antiviral function, these cytokines play an important role in the induction of cytokine expression in surrounding tissue ( Kawai & Akira 2006 ). In summary, showed up with the exception of IFN-β expression in HaCaT cells, a higher induction of the expression of type-I-IFN after transduction with 1.1 × 10^10^ IU of Ad-GFP transfection compared with an equivalent amount of DNA. Taking account of the transduction and transfection efficiency the data demonstrates the immunogenic potential of adenoviral DNA in keratinocytes (Figure 1D).

### Evaluation of cytokine expression after adenoviral DNA internalization

In order to get a deeper insight into cytokine expression after adenoviral DNA internalization into keratinocytes, an enlarged number of samples was analyzed. For this purpose the data from tests on different days with cells of different passages (HaCaT) and different patients (HKC) were examined at different time-points (type-I-IFN: n = 18 samples; cytokines: n = 6 samples).

The data (Figure 1E) showed a 1.7-fold increased expression of IFN-α in HaCaT cells (p = 0.0836) and 1.5-fold in HKC (p = 0.3329). For the expression of IFN-β mRNA a 26.1-fold, significant increase in HaCaT cells (p = 0.0029) could be detected. Transfection of primary keratinocytes, however, resulted in a 11.4-fold increase (p = 0.0439). The study showed an induction of cytokine gene expression of IL-1α and IL-6 in HaCaT cells by a factor of 10.3 (IL-1α; p = 0.0972) and 22.1 (IL-6; p = 0.0074) respectively. In contrast, the induction of IL-1α (2.6-fold; p = 0.0233) and IL-6 (2.1-fold; p = 0.0566) in HKC was comparatively lower. In addition to the induction of interferons and interleukins showed the transfection of keratinocytes also a significant increase of IL-8-encoding mRNA. In HaCaT cells, a 11.9-fold (p = 0.0038) induction has been detected whereas HKC possessed a 6.9-fold induction (p = 0.0312). The expression of TNFα showed mean values of 4.9-fold induction in HaCaT cells (p = 0.0478) and 1.6-fold increase in HKC (p = 0.5123).

### Adenovirally induced immune reaction in *ex vivo* cultivated human full-thickness skin

**Figure 2:**
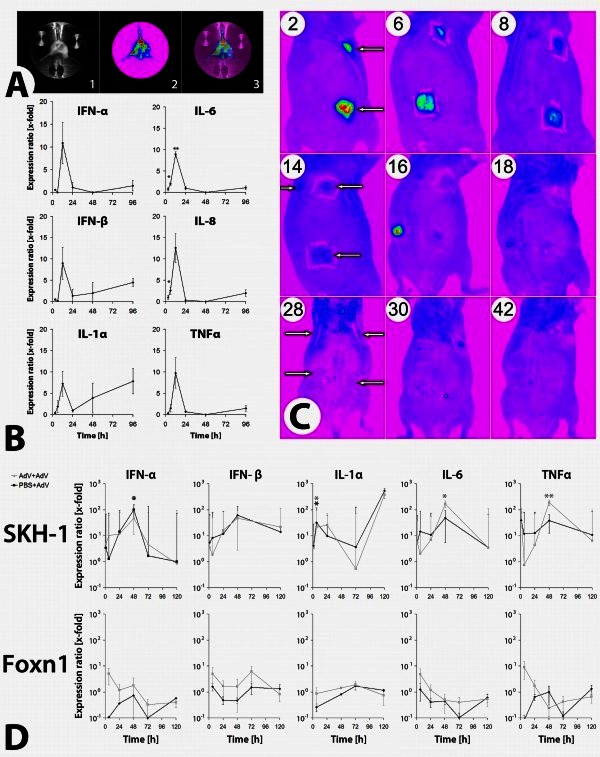
**Immune reaction after cutaneous adenoviral gene delivery.** (**A**) Transduction control of a Bo-Drum®. Representative exposition of a Bo-Drum® 48 h after transduction of fixed skin sample with 1 × 10^10^ IU GFP-encoding adenoviral vector (Ad-GFP) (1: transmitted light image; 2: fluorescent image; 3: overlay). (**B**) Kinetics of cytokine expression after Bo-Drum® transduction. Time course (3 - 96 h) of cytokine mRNA expression (x-fold in relation to a vehicle control) after *ex vivo* transduction of human full skin in relation to a vehicle control (PBS injection). Per timepoint, n = 6 samples (from two different patients) were transduced with 1 × 10^10^ IU Ad-GFP (* = p < 0,05; ** = p < 0,005). (**C**) Adenovirus induced immune reaction *in vivo*. GFP-fluorescence detection of immunocompetent, hairless mice (SKH-1^h/r^) at timepoints of 7–42 days after intradermal injection (white arrow) of 10^10^ IU Ad-GFP (n = 3 per group). An additional vector application and reapplication of the same vector doses was performed on day 14 and 28 (* = p < 0.05, ** = p < 0.005). (**D**) Type-I-interferon and cytokine expression *in vivo*. RT-PCR analysis of type-I-interferon and cytokine expression at timepoints of 1, 6, 24, 48, 72 and 120 h after first application (PBS + AdV) and reapplication (AdV + AdV) of 10^10^ IU Ad-GFP (* = p < 0.05, # = p < 0.005; AdV + AdV) of 10^10^ IU Ad-GFP in immunocompetent (SKH-1^h/r^) (A) and athymic (Foxn-1^nu^) (B) mice. Data was presented as mean ± SEM (n = 3 per group/timepoint).

Since keratinocytes in a physiologic full skin environment may react differently from cultured cells, human full skin explants (n = 6 samples) were intradermally transduced *ex vivo* with an adenovirus type 5 vector (Ad-GFP) (Figure 2A). RT-PCR analysis (Figure 2B) showed an induction of pro-inflammatory cytokines peaking 12 h post transduction (IFN-α: 11-fold; p = 0.163); IFN-β: 9-fold, p = 0.168); IL-1α: 7-fold, p = 0.167); IL-6: 9-fold, p = 0.001); IL-8: 13 -fold, p = 0.076); TNFα: 10-fold, p = 0.133)). A re-increase in expression of IFN-β (4.5-fold, p = 0.05) and IL-1α (7.8-fold, p = 0.137) could be observed after 96 h.

Since there is a lack of systemic influences by using the human *ex vivo* full skin transduction for an investigation of adenovirus induced systemic immune reactions, the aim of our *in vivo* study was gaining a deeper insight into systemic influences towards adenovirus induced inflammatory response in a murine skin transduction model.

### Adenovirally induced immune reaction in an *in vivo* murine transduction model

In our *in vivo* study, an application of 10^10^ IU Ad-GFP resulted in strong GFP expression with a peak at day 2 and remained detectable for 12 days (Figure 2C). Reapplication of the same dose of Ad-GFP on days 14 and 28 led to a reduced in intensity and duration (5 days) of GFP fluorescence.

In a second experiment, immonucompetent (SKH-1^hr^) and athymic (Foxn-1^nu^) mice were intradermally transduced with 10^10^ IU Ad-GFP on day 0 followed by a retransduction with the same vector dose into the same (AdV + AdV) and two new areas (PBS + AdV) on day 14. RT-PCR analysis of skin samples from immunocompetent mice showed an induction of IFN-α/β, IL-6, IL-10 and TNF-α 24 to 48 h post transduction (Figure 2D). RT-PCR analysis of samples from athymic mice exhibited a faster cytokine induction which was, however, on a significantly lower level when compared to immunocompetent mice.

### DNA recognition and signal transduction

An induction of innate immunity requires different key molecules for inflammatory signal transduction, including MAPK-dependent signaling pathways (Erk2, MAPKK, p38 MAPK), JNK, JAK/STAT-cascades and the transcription factor NFκB. These factors represent key molecules of the toll-like receptor signaling pathway, but they can also interact with members of the RIG-like receptor family or the DNA-binding receptor DAI ( Akira & Hoshino 2003 ; Kanehisa 2012 ; Yoneyama & Fujita 2007 ). In contrast to p38 MAPK, JNK and NFκB, an activation of Erk2 has not been described for the RIG-like receptor and DAI pathway ( Kanehisa 2012 ).

**Figure 3:**
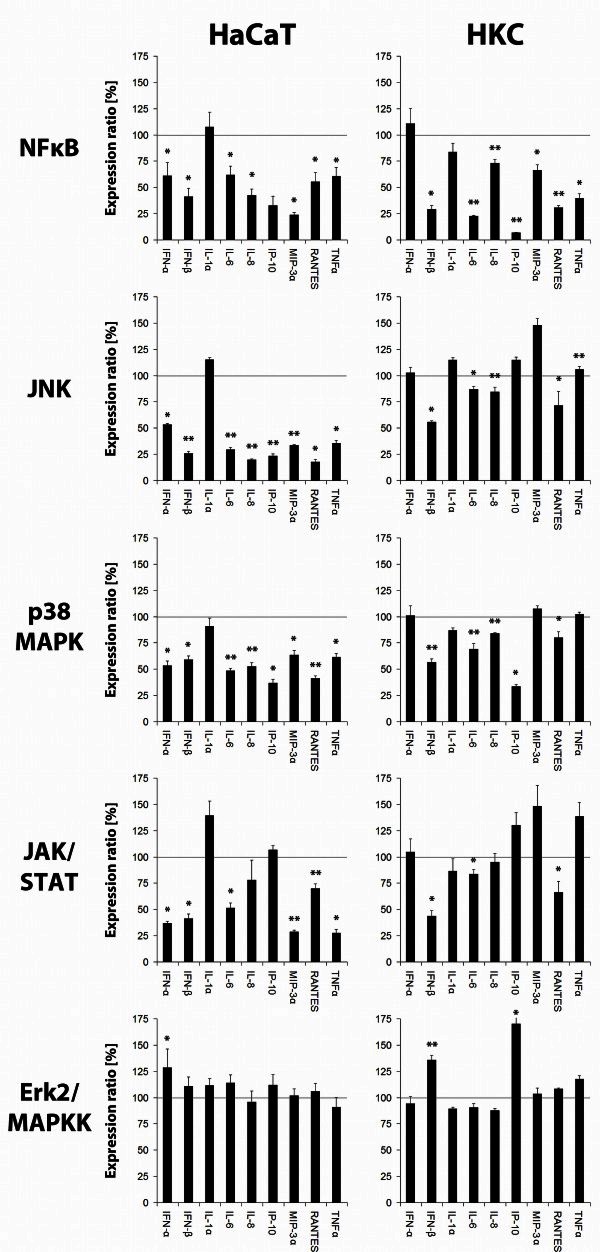
**Inhibition of signaling cascades.** Cytokine expression after transfection of HaCaT cells and HKC with 5 μg/ml of adenoviral DNA with a follow-up of 15 h (HaCaT) or 6 h (HKC). One hour before transfection, inhibitors of indicated signaling molecules were added to the cell culture medium (10 mM final concentration). Data is indicated as percentage expression of cytokines in relation to a non-inhibited positive control (* = p <0.05, ** = p <0.005).

The following section describes the data of a further stimulation of keratinocytes using specific inhibitors. This showed a predominantly significant reduction of cytokine expression after inhibition of NFκB, JNK, p38 MAPK and JAK/STAT in HaCaT cells and HKC (Figure 3). In contrast, inhibition of Erk2 did not show any significant reduction of cytokine expression. This data also represents an indication for induction of cytokine expression by RIG-like receptors.

### Expression of nucleic acid sensing receptors after adenoviral DNA delivery

**Figure 4:**
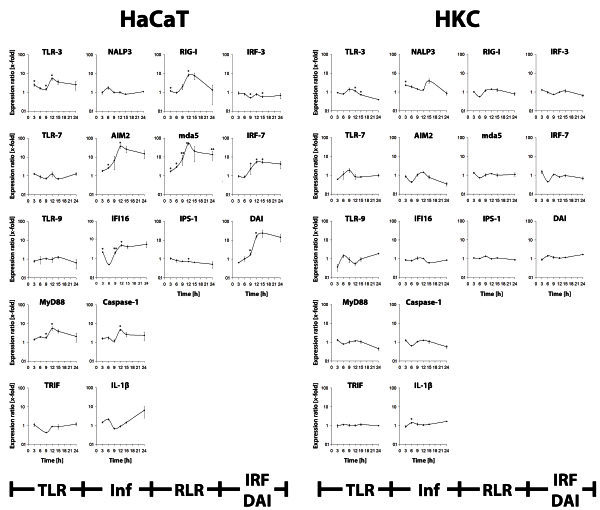
**Induction of innate immune signalling cascades.** Induction of Toll-like receptors (TLR), Inflammasome-building components (Inf), RIG-like receptors (RLR), Interferon-regulatory factors (IRF) and DAI expression (x-fold in relation to a vehicle control) 15 h (HaCaT) or 6 h (HKC) after transfection of HaCaT cells and HKC with 5 μg/ml of adenoviral DNA (* = p <0.05, ** = p <0.005).

For an examination of the involvement of TLRs, RLRs and DAI, an analysis of the expression profiles of these receptors and specific adapter molecules using qRT-PCR has been performed (Figure 4). In HaCaT cells, highest induction of mRNA expression after transfection was measured for mda5 (52.08-fold; 12 h post trasfection (p = 0.0267)), DAI (23.86-fold; 15 h post transfection (p = 0.1349)) and AIM2 (36.81; 12 h post transfection (p = 0.0421)) whereas HKC showed a significant increase in the NALP3 mRNA (3.29-fold, p = 0.0243) only 3 h post transfection.

The data of the strong induction of AIM2 expression in HaCaT cells suggests a key role of this receptor in eliciting an anti-adenoviral immune reaction. Furthermore, the expression of NALP3 in HKC was one of pronounced increase in this cell type and may therefore constitute a key role in the detection of adenoviral DNA. Additionally, an involvement of IFI16 could not be excluded. Due to the comparably lower regulation of IFI16 mRNA expression, further analysis focused on the factors AIM2, NALP3, mda5 and DAI.

### Recognition of adenoviral DNA in keratinocytes

For a more detailed statement about the involvement of specific receptors on the adenoviral-induced immune response, the expression of receptors AIM2, NALP3, mda5 and DAI was inhibited in a further experiment using siRNA over a period of 48 hours. Hence, the cells were transfected with adenoviral DNA (5 μg/ml medium), and the impact on interferon expression was determined by qRT-PCR.

**Figure 5:**
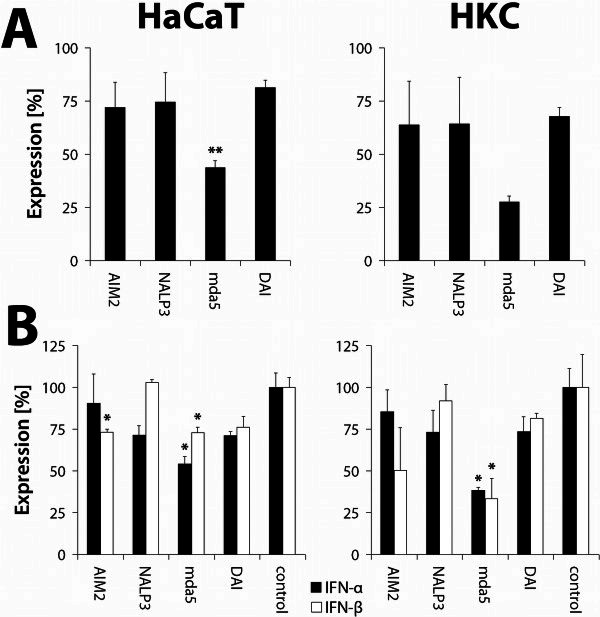
**siRNA-mediated inhibition of gene expression.** (**A**) Efficiency of siRNA - mediated inhibition of gene expression. Expression of AIM2, NALP3, mda5 and DAI mRNA in HaCaT cells and HKC 48 h after siRNA transfection (* = p <0.05, ** = p <0.005). (**B**) Effects of siRNA - mediated inhibition of potential receptor gene expression. Expression of type-I-interferon (x-fold in relation to a vehicle control) after siRNA-mediated inhibition of potential DNA receptor mRNA expression. Two days after silencing, cells were transfected with 5 μg/ml isolated adenoviral DNA for 15 h (HaCaT) or 6 h (HKC) (* = p <0.05).

To check the efficiency of the used siRNA-treated cells were analyzed for expression of the inhibited receptors (Figure 5A). All treated samples showed a reduction of corresponding mRNA expression. The efficiency of anti-AIM2-siRNA (siAIM2) showed a residual expression of the mRNA of 71.89% (p = 0.1404) compared with a control in HaCaT cells and 63.79% (p = 0.4377) in HKC. The inhibition of NALP3 expression led to a residual expression of 74.54% (HaCaT, p = 0.2023) and 64.25% (HKC, p = 0.1987). The highest efficiency was observed in siMDA5 treated samples (HaCaT: 43.72%, p = 0.0035; HKC: 27.48%, p = 0.0572). In siDAI treated samples, a residual mRNA expression of 81.38% (HaCaT, p = 0.4675) and 67.75% (HKC, p = 0.0923) was detected.

Analysis of type-I-IFN expression of siMDA5 treated samples offered a significant reduction in expression of IFN-α and IFN-β in both HaCaT cells and HKC (Figure 5B). The largest effect on interferon expression was observed in primary keratinocytes (IFN-α: 38.31%, p = 0.0333), IFN-β: 33.47%, p = 0.0569). In HaCaT cells, a residual expression of 54.11% (IFN-α, p = 0.0180) and 72.99% (IFN-β, p = 0.0262) was measured.

In case of siAIM2 treated samples, a significantly greater reduction of IFN-β (HaCaT: 73.11%, p = 0.0355; HKC: 50.23%, p = 0.2050) compared to IFN-α (HaCaT: 90.57%, p = 0.7026; HKC: 85.30%, p = 0.5735) was shown. In contrast, the siNALP3 treated samples possessed a greater reduction of IFN-α (HaCaT: 57.58%, p = 0.0586; HKC: 73.36%, p = 0.2570) compared to IFN-β (HaCaT: 103.99%, p = 0.3560; HKC: 92.03%, p = 0.7426).

The treatment of keratinocytes with siDAI showed a matching expression of IFN-α and IFN-β for both cell types. In HaCaT cells, a residual expression of 71.22% (IFN-α, p = 0.0690) and 76.15% (IFN-β, p = 0.0546) was measured. In HKC, an expression of 73.56% (IFN-α, p = 0.1962) and 81.54% (IFN-β, p = 0.4496) were determined.

For an accurate interpretation, the results of type-I-IFN expression in different samples were normalized to the corresponding siRNA efficiency (Table 3). The data generated here will give a closer insight into the role of individual receptors in the induction of immune response. The ratio of siRNA efficiency and type-I-IFN expression correlates with the importance of each receptor in the induction of the immune system, meaning a higher ratio indicates a greater participation in eliciting an innate immune response.

**Table 3:** **Normalized data of type-I-interferon expression after siRNA - mediated inhibition of gene expression of potential DNA receptors in HaCaT cells and HKC ([siRNA - Efficiency (Expression in % of control)/type-I-IFN expression (Expression in % of control)] ± SEM)**

	AIM2		NALP3		mda5		DAI	
	***HaCaT***	***HKC***	***HaCaT***	***HKC***	***HaCaT***	***HKC***	***HaCaT***	***HKC***
**IFN-alpha**	0,79±	0,75±	1,29±	0,88±	0,81±	0,71±	1,14±	0,92±
	0,15	0,12	0,12	0,15	0,07	0,03	0,04	0,11
**IFN-beta**	0,98±	1,27±	0,72±	0,70±	0,60±	0,82±	1,07±	0,83±
	0,02	0,65	0,01	0,07	0,03	0,3	0,09	0,03

For this, AIM2 plays an important role in the induction of IFN-β. In contrast, NALP3, however causes a stronger induction of IFN-α. DAI plays a major role in the induction of both IFN-α and IFN-β whereas the RNA-binding receptor mda5 also plays a role in type-I-IFN induction, which, however, is lower when compared to AIM2, NALP3 and DAI.

### Vector efficacy after inhibition of potential DNA - receptors

In order to investigate the effects of siRNA - mediated gene silencing on efficacy of transgene expression, HaCaT cells were incubated with previously mentioned siRNAs over a period of 48 hours and further transduced with 1 × 10^8^ IU of Ad5-GFP. After another 48 hours, the reporter fluorescence was quantified using a kodak life-imaging station.

**Figure 6:**
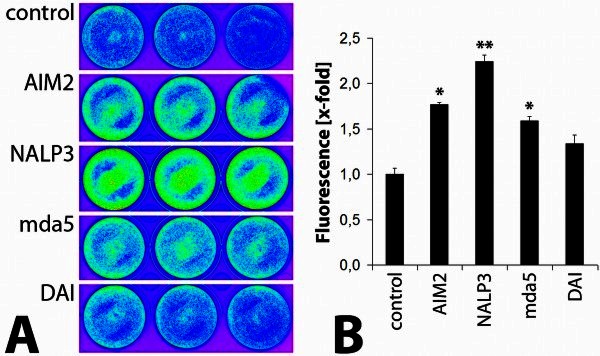
**Transgene expression after siRNA-mediated gene expression of AIM2, NALP3, mda5 and DAI.** Quantitative (**A**) and qualitative (**B**) representation of the GFP expression in HaCaT cells 48 h after siRNA - mediated inhibition of AIM2, NALP3, mda5 and DAI and subsequent transduction with 1 × 10^8^ IU Ad5-GFP or PBS (control) (* = p < 0.05; ** = p < 0.005).

Qualitative data analysis of the transduced keratinocytes (Figure 6A) possessed a stronger transgene fluorescence in cells treated with siRNA targeting AIM2, NALP3, mda5 and DAI. Quantitative analysis of GFP fluorescence (Figure 6B) illustrated a 2.24-fold, significantly increased after inhibition of NALP3 (p = 0.0018). Pre-incubation with anti-AIM2-siRNA and anti-mda5 siRNA also leads to a significant increase in reporter fluorescence by a factor of 1.77 (AIM2, p = 0.0186) and 1.59 (mda5, p = 0.0244). A 1.34-fold amplified signal intensity was found after using anti-DAI-siRNA (p = 0.0902).

Hence, the data generated in this experiment indicates a primary involvement of the receptors AIM2, NALP3 and mda5 but also DAI in the induction of the innate immune response after adenoviral gene transfer keratinocytes.

## Discussion

There are several studies that examine adenovirally induced innate immune reactions in mice, primates and humans ( Raper et al. 2003 ; Schnell et al. 2001 ; Zhang et al. 2001 ). However, these studies have a propensity to only address specialized APCs such as DCs or MΦ ( Nociari et al. 2007 ; Zhu et al. 2007 ) and RNA virus-induced immune reactions of APC ( Diebold et al. 2004 ). In addition, data on the role of keratinocytes in innate immunity, particularly towards DNA internalization and DNA virus infection is limited. For optimization of adenoviral gene delivery into skin it is critical to shed light on the molecular mechanisms of immunity of epidermal cells. To address these issues, we have investigated the role of keratinocytes in innate immunity in response to DNA internalization.

This study used the HaCaT cell line as it is simple to cultivate and experiments were carried out under highly standardized and reproducible conditions ( Boukamp et al. 1988 ). The data of constitutive cytokine expression in HaCaT cells displayed expected differences in comparison to HKC. However, these relate primarily to differences in expression levels of these factors, while all measured factors were expressed in both cell types. Consistent with Köllisch et al., HKC and HaCaT cells also revealed generally comparable data for induction of inflammatory factors after transfection of adenoviral DNA ( Köllisch et al. 2005 ). In comparing both cell types, a difference in induction intensity and time response of these inflammatory factors has been detected in a previous study and could be confirmed by our experiments. However, HaCaT cells possessed a higher cytokine induction than HKC whereas HKC exhibited a faster reaction towards adenoviral DNA and might be caused by different factors like genetic abberations described for HaCaT cells and due to comparing highly proliferative HaCaT cell line and predominantly terminal differentiated HKCs ( Steinstraesser et al. 2011 ).

In order to determine the differences in transfection and transduction efficacy of keratinocytes, cells were stimulated isolated adenoviral DNA or an equal dose of adenoviral vectors. The transduction efficacy of adenoviral vectors was 83% for HaCaT and 92% for HKC respectively, while transfection of keratinocytes with an equivalent dose of adenoviral DNA possessed a significant lower efficacy (HaCaT: 15%; HKC: 9%). In regard to transfection and transduction efficacy, transfection revealed a type-I-IFN expression on a comparative level as measured after transduction of keratinocytes and points out an important role of adenoviral DNA in the induction of innate immune response in keratinocytes.

As discussed above, recognition of cytoplasmatic localized viral DNA triggers the antiviral innate immune response. Studies have described an activation of innate immunity independent of the species and sequence of the internalized DNA; the induction of type-I-interferon and cytokine synthesis during introduction of nucleic acids into the cytoplasm of APCs has been shown by different groups ( Ishii et al. 2001 ; Yasuda et al. 2005 ). Data regarding immunogenicity of cytoplasmatic localized DNA in epidermal cells is limited. For this purpose, this study demonstrates a high induction of IFN-α/β, pro-inflammatory cytokines and chemokines by adenoviral DNA internalization *in vitro, ex vivo* and *in vivo* ( Steinstraesser et al. 2011 ).

Obtained *in vivo* data suggests immunization against adenovirus, which led to decreased intensity and duration of transgene expression after reapplication of the identical vector dose into the same or non-transduced areas in immuncompetent mice. Therefore, therapeutic reapplication of the vector into keratinocytes to stabilize transgene expression may be unefficient ( Nociari et al. 2007 ; Zhu et al. 2007 ). Moreover, athymic mice showed a diminished type-I-IFN and cytokine expression (Figure 2D) and a stable GFP level starting from day 5 ( Steinstraesser et al. 2011 ), suggesting the possibility to optimize adenoviral gene delivery by modulating the inflammatory responses of the skin. An early decrease in GFP level detected in immunocompetent and athymic mice from day 2 to 5 denotes a T-cell independent mechanism for a reduction of GFP level ( Steinstraesser et al. 2011 ) and was dedicated to an induction of innate immunity ( Muruve 2004 ). Moreover, data from athymic mice and *in vitro* stimulation of keratinocytes suggests an important role of keratinocytes in eliciting an innate immune reaction after adenoviral challenge.

In previous years, many studies regarding the molecular mechanisms in DNA recognition were evaluated for the stimulation of different cell types with various species of DNA. For example, several groups have shown a major role of TLRs, the DNA - binding protein DAI as well as special forms of the inflammasome (AIM2, NALP3, IFI16) in macrophages, dendritic cells and fibroblasts ( Goubau et al. 2010 ; Nociari et al. 2007 ; Takaoka et al. 2007 ; Unterholzner et al. 2010 ; Zhu et al. 2007 ).

However, our current understanding of the signal transduction after recognition of adenoviral DNA in keratinocytes is still rather limited. For the establishment of cutaneous adenoviral gene delivery, information about immune reactions towards adenoviral DNA is of high importance.

The data from this study depicted a special role of the JAK/STAT signaling pathway in induction of type-I-IFN. Since JAK/STAT plays a major role in signal transduction of type-I-IFN- and cytokinereceptors, the involvement of the JAK/STAT signaling pathway in antiviral immunity was not unexpected.

Additionally, NFκB, JNK and p38 MAPK but not Erk2 were also characterized as important factors in adenovirally induced signal transduction. The proteins NFκB, p38 MAPK and JNK play an important role not only in the TLR mediated signal transduction but also in RLR-dependent (RIG-I and mda5) signaling ( Kanehisa 2012 ). Since Erk2/MAPKK are not addressed by the TLR pathway, the lack of participation of Erk2/MAPKK in activation of type-I-IFN expression suggests an RLR-dependent pathway for adenoviral DNA recognition ( Kanehisa 2012 ).

In analyzing potentially in adenovirus recognition involved receptor systems, a significant induction of mda5 was observed. An activation of RLRs is associated with an involvement of IRFs, NFκB, p38 MAPK and JNK, confirming the previous results of the inhibitor study. Since a signal transduction occurs via RLRs, which do not address Erk2/MAPKK, thus would explain the results of the type-I-IFN expression after inhibition of Erk2/MAPKK.

Additionally, there was also an induction of mRNA expression of NALP3- and AIM2 inflammasomes. Hence, a significant induction of inflammasome-activated caspase-1 and IL-1β, which is processed by caspase-1, was shown for both cell types. This fact suggests an involvement of inflammasomes in the adenovirally induced immune response.

In addition to the previously detected increased expressions of AIM2, NALP3 and mda5, the analysis of the DAI expression possessed a significant increase in HaCaT cells. This also indicates an involvement of this protein out of the adenovirally induced immune response and may also explain the NFκB dependence seen in the inhibitor study.

Since an analysis of keratinocytes derived from expression profiles only permits a tendency of the molecular mechanisms of immune induction. A firm statement concerning the participation of the above mentioned receptors, however, can only be provided by targeted inhibition of these factors. Therefore, potential receptor molecules that were identified in the preliminary screening, were silenced using siRNA in order to get a closer information about their involvement in recognition of adenoviral DNA.

Considering the data of siRNA efficacy, the greatest effect of type-I-IFN expression was measured in keratinocytes with reduced expression of AIM2, NALP3 and DAI. On this occasion NALP3- and AIM2-inhibited samples possessed an opposite trend in induction of interferons: Silencing of NALP3 had a greater effect on IFN-α expression and silencing of AIM2 a greater effect on IFN-β expression. In case of transfection of keratinocytes with adenoviral DNA, mda5 plays also role in induction of innate immune reaction.

An additionally performed transduction of keratinocytes treated with siRNA blocking AIM2, NALP3, DAI and mda5 gene expression possessed an increase in GFP protein level. Interestingly, the experiments carried out a participation of RLRs, particularly mda5 in adenovirus recognition. The activation of p38 MAPK, JNK, and NFκB, but not Erk2, which has been reported in this study, clearly supports this thesis ( Kanehisa 2012 ).

Since mda5 has been described as a RNA recognizing receptor, it should principally not be involved in adenovirus recognition ( Kawai & Akira 2008 ). In the literature, however, another publication concerning a mda5-dependent induction of an immune response after infection of monocytes with a DNA virus (vaccinia virus modified Ankara, MVA) was found ( Delaloye et al. 2009 ). The exact mechanism of mda5-induced immune response could not be clarified in that study. One possible explanation for this might be a detection of transcripts of AT-rich DNA sequences in the cytoplasm ( Bauernfeind et al. 2010 ). These are encoded by the late regions of the adenoviral genome, are responsible for inhibiting the activity of protein kinase R after infection of a cell responsible and are produced in quantities of 10^8^ copies per cell ( Acheson 2011 ). This also underlines the thesis of RLR-induced induction of the immune response in keratinocytes.

Although the science of cutaneous adenoviral gene delivery is complex and riddled into many isolated experiments. Since knowledge about signal transduction of innate immune reaction after adenoviral gene delivery into skin is very limited, data about an involvement of AIM2, NALP3, DAI and mda5 in detection of adenoviral DNA adds new features in our understanding of current scopes and future opportunities of cutaneous gene therapy using adenoviral vector systems. Further studies are needed to improve our knowledge in molecular mechanisms of signal transduction in adenovirus induced immune reactions of the skin. Their contribution remains vital to continue to search for clues, yet their exact role in the overall context in molecular mechanisms in innate immunity of the skin is still to be understood.

### Ethical standards

The authors declare that the experiments comply with the current laws of germany.
